# Tauroursodeoxycholic acid: a potential therapeutic tool in neurodegenerative diseases

**DOI:** 10.1186/s40035-022-00307-z

**Published:** 2022-06-04

**Authors:** Kareem Khalaf, Paolo Tornese, Antoniangela Cocco, Alberto Albanese

**Affiliations:** Department of Neurology, Humanitas Research Hospital, Rozzano, MI Italy

**Keywords:** Amyotrophic lateral sclerosis, Alzheimer’s disease, Bile acids, Disease-modifying, Huntington’s disease, Neurodegeneration, Neuroprotection, Parkinson’s disease, Ursodeoxycholic acid, Tauroursodeoxycholic acid

## Abstract

Most neurodegenerative disorders are diseases of protein homeostasis, with misfolded aggregates accumulating. The neurodegenerative process is mediated by numerous metabolic pathways, most of which lead to apoptosis. In recent years, hydrophilic bile acids, particularly tauroursodeoxycholic acid (TUDCA), have shown important anti-apoptotic and neuroprotective activities, with numerous experimental and clinical evidence suggesting their possible therapeutic use as disease-modifiers in neurodegenerative diseases. Experimental evidence on the mechanisms underlying TUDCA’s neuroprotective action derives from animal models of Alzheimer’s disease, Parkinson’s disease, Huntington’s diseases, amyotrophic lateral sclerosis (ALS) and cerebral ischemia. Preclinical studies indicate that TUDCA exerts its effects not only by regulating and inhibiting the apoptotic cascade, but also by reducing oxidative stress, protecting the mitochondria, producing an anti-neuroinflammatory action, and acting as a chemical chaperone to maintain the stability and correct folding of proteins. Furthermore, data from phase II clinical trials have shown TUDCA to be safe and a potential disease-modifier in ALS. ALS is the first neurodegenerative disease being treated with hydrophilic bile acids. While further clinical evidence is being accumulated for the other diseases, TUDCA stands as a promising treatment for neurodegenerative diseases.

## Introduction

Neurodegenerative diseases are characterized by the progressive deterioration of neuronal function, ultimately leading to a loss of specific neurons. These are incurable diseases, and current available therapies, at best, only manage clinical symptoms. Although the pathological hallmarks and the affected neuronal populations can vary, when considered at the genetic, molecular, or cellular level, relatively few players and patterns crop up repeatedly, such as the aggregation and spread of misfolded proteins, selective vulnerability of particular neurons, and activation of immune responses [1, 2]. The possibility that such pathological phenomena arise from common mechanisms that play out across different brain regions and cell types, or simply as the same steps along a shared pathway to neurodegeneration, raises hope for finding treatments that modify the disease course of neurodegenerative diseases.

The peculiar anatomical specificity of neuronal degeneration characterizes the profile of neurodegenerative diseases. An early pathological feature in Alzheimer’s disease (AD) is the degeneration of cholinergic neurons in the subcortical nuclei of the basal forebrain; in Parkinson’s disease (PD) degeneration of dopaminergic neurons occurs in the substantia nigra pars compacta. Huntington’s disease (HD), instead, is characterized by selective neuronal loss in the striatum; in amyotrophic lateral sclerosis (ALS) degeneration prevalently affects corticospinal and spinomuscular motor neurons. The progression of neurodegeneration varies considerably from a few years to several decades in different diseases. ALS is the most fast-progressing neurodegenerative condition, with survival varying from 2 to 4 years from onset [3]. Other neurodegenerative conditions have a slower course, although with significant individual variations in progression trajectories [4].

Stimuli that trigger the onset of metabolic derangement and lead to neuronal death include reactive oxygen species (ROS) production, misfolded protein accumulation, and endoplasmic reticulum (ER) stress. These stimuli are kept in check by mechanisms protecting the cell, such as the survival pathways [5]. Since the pathways involved in neuronal death are common to different neurodegenerative diseases, it is believed that modulating these may be beneficial against different disorders.

**Fig. 1:**
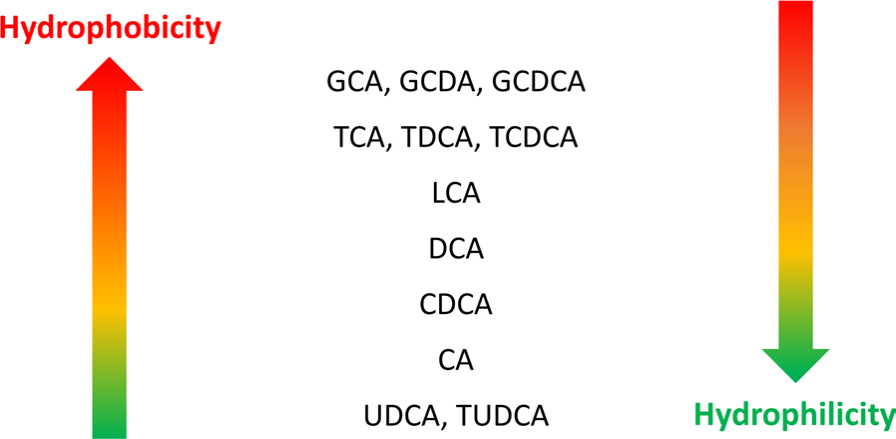
Bile acids can be differentiated based on their polarity, as summarised by this figure. More hydrophobic bile acids are represented by their acronyms in the upper part, whereas more hydrophilic are in the bottom part. Abbreviations: CA, cholic acid; CDCA, chenodeoxycholic acid; DCA, deoxycholic acid; GCA, glycocholic acid; GCDCA, glycochenodeoxycholic acid; GDCA, glycodeoxycholic acid; LCA, lithocholic acid; TCA, taurocholic acid; TCDCA, taurochenodeoxycholic acid; TDCA, taurodeoxycholic acid; TUDCA, tauroursodeoxycholic acid; UDCA, ursodeoxycholic acid. Modified from [12]

Bile acids are one emerging therapy to counteract cellular programmed death pathways in neurons. These hydroxylated steroids are synthetized in the liver from cholesterol. Peroxisomal enzymes assist in the hepatic biosynthesis of bile acids. They are normally conjugated to glycine and taurine, or sulphated in the liver. The active transport of bile acids across canalicular membranes of hepatocytes is a primary driving force for bile flow [6]. The hydrophobicity of bile acids decreases with an increase in hydroxyl groups. Ursodeoxycholic acid (UDCA) and tauro-ursodeoxycholic acid (TUDCA) are among the most hydrophilic bile acids [7]. The degree of hydrophobicity of the various bile conjugates is outlined in Fig. 1. Due to their hypothesized anti-apoptotic action, hydrophilic bile acids, specifically TUDCA, are considered potential therapeutic tools for neurodegenerative diseases. Importantly, UDCA and TUDCA can cross the blood–brain barrier (BBB) [8–10] and exert protective effects in the brain [11].

We report here the current knowledge on the potential therapeutic action of hydrophilic bile acids in neurodegenerative conditions, focusing in particular on TUDCA, the most hydrophilic among bile acids. We searched PubMed for animal studies, clinical trials, reviews, systematic reviews and meta-analysis on hydrophilic bile acids, using a variable combination of the terms “TUDCA”, “UDCA”, “hydrophilic bile acids”, “neuroprotection” or “neurodegenerative disease”, with no filtered restrictions. We identified published studies prior to the writing process, and cited papers of relevance to our aim to review the current knowledge on the potential therapeutic action of hydrophilic bile acids in neurodegenerative conditions. We searched ClinicalTrials.gov for any past and ongoing trials on hydrophilic bile acids in neurodegenerative diseases, to report for future perspectives.

## Neuroprotection and disease modification

The majority of neurodegenerative disorders are proteinopathies. Impaired protein homeostasis causes proteins to misfold and accumulate in aggregates [1, 13]. Understanding the molecular and biochemical pathogenesis of neurodegenerative diseases is essential for the discovery of neuroprotective therapies that aim to achieve disease modification. Pathways that represent suitable targets for intervention require compounds that target one or multiple factors in that pathway. These compounds need to be tested in specific animal models before being assessed in clinical trials suitably designed to demonstrate a disease-modifying effect. In addition to clinical variables, study endpoints can measure a wide range of disease-specific markers, such as amyloid-β, tau, α-synuclein, neurogranin, or neurofilaments [14, 15].

Neuroprotection indicates the capability to reduce, halt, or reverse neurodegeneration at the cellular level. Disease modification indicates the clinical evidence to delay a meaningful endpoint in a properly run clinical trial. Putatively neuroprotective drugs are the logical candidates for disease-modification trials, to provide the evidence needed for regulatory approval of innovative medications. A properly active neuroprotective compound that modulates the relevant cellular mechanisms involved in neurodegeneration might not be able to modify the disease clinical course, as demonstrated by clinical trials [16]. There are several possible explanations for this prospect. Therapies might have an effect on the targeted cellular and molecular mechanisms, but long-lasting maintenance of neuronal function might not be achieved [17]. A further possibility is that neuroprotective treatment is started too late after the prodromal stage, when recovery of neuronal homeostasis is no longer achievable. Another possible reason is the experimental nature of animal disease models. A range of experimental models based on genetic and environmental factors are currently available to help identify a potential therapeutic target. However, these models do not necessarily reproduce the complex and progressive nature of the neurodegenerative human pathology [18].

There are therefore several possible explanations for negative disease-modification trials. Experimental research has highlighted many potential pathways of neuronal cell death, including excitotoxicity, oxidative damage by ROS, necrosis, and glial injury, which have therapeutic potential in neurodegenerative diseases. Disease-modifying agents that act on these pathways are under evaluation [19, 20]. It is envisaged that future disease-modifying medications will intervene on these pathways, although it is still unsettled whether single or multiple interventions are appropriate.

Symptomatic therapies that attenuate symptoms are available only for a few neurodegenerative disorders. The case of PD shows the extent to which symptomatic therapies can dramatically help patients, improving their quality of life, independence and employability, although they mostly fail to modify disease progression at the cellular level [16]. Furthermore, it has been proposed that early initiation of a symptomatic therapy in PD may restore basal ganglia physiology, thereby preventing or delaying disease complications [21]. The possibility that symptomatic treatments can also exert a disease-modifying influence, without being specifically neuroprotective to neurons, is a topic of debate. Two prospective double-blind clinical trials have tested the capacity of dopamine agonists to modify the rate of PD progression in newly diagnosed patients, using imaging markers as primary endpoints [22, 23]. The Early versus Late levodopa in PD study investigated the effect of levodopa versus placebo on disease progression [24]. It is likely that in PD, early initiation of effective symptomatic treatments may normalize basal ganglia functioning and improve the prognosis of individual patients [21]. While available data indicate that early initiation of symptomatic medications has a positive effect on disease course, the quest for disease-modifying drugs with specific neuroprotective action remains open.

Ultimately, a disease-modifying effect is expected to target the specific pathways involved in neurodegenerative diseases, providing plausible understanding of therapeutic efficacy that is not simply symptomatic.

## Hydrophilic bile acids in neurodegenerative diseases

**Fig. 2:**
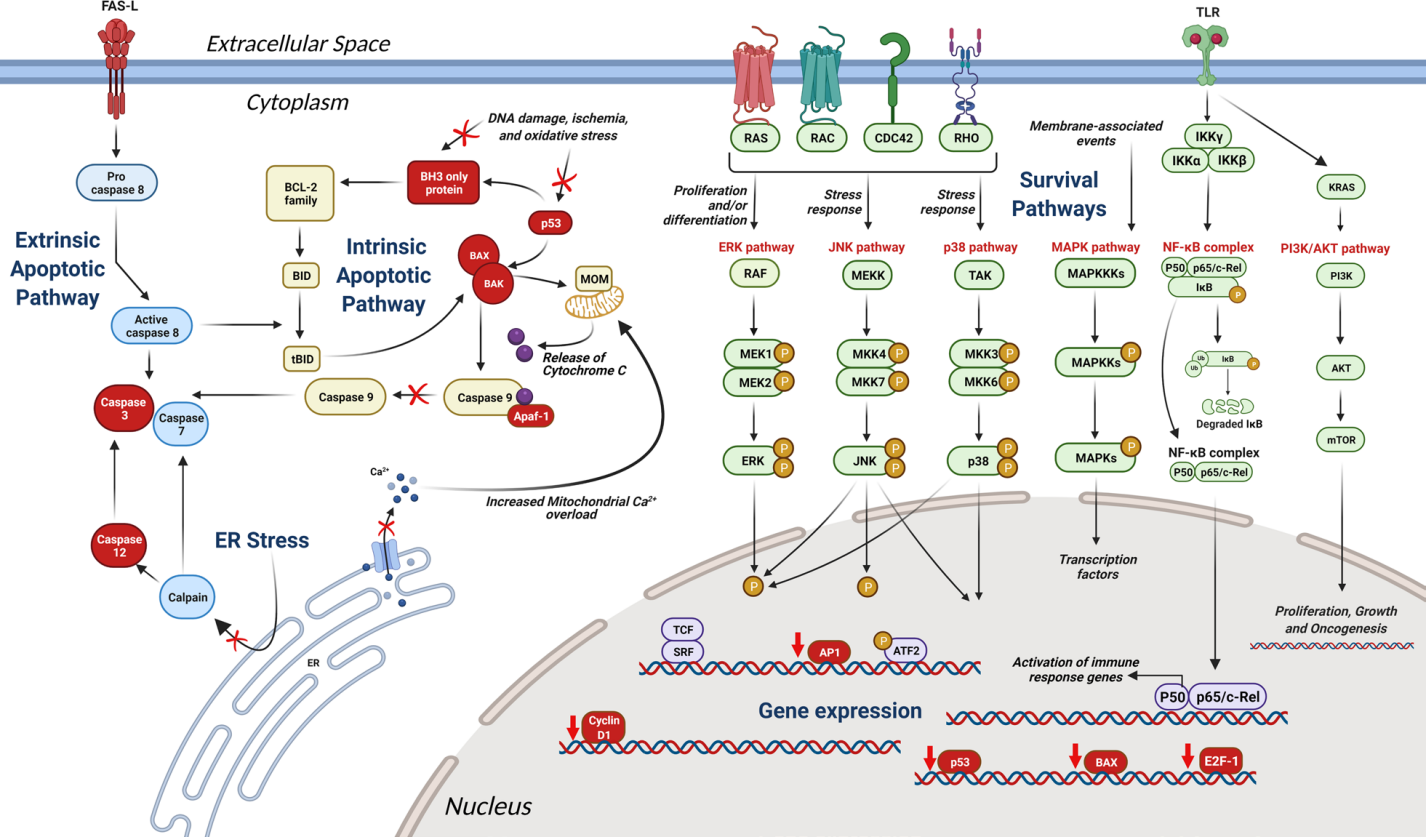
Schematic drawing of different possible neuroprotective mechanisms exerted by hydrophilic bile acids, with specific reference to the anti-apoptotic effects of TUDCA on different intracellular pathways. Factors and pathways inhibited by TUDCA are shown in red; pathways blocked by TUDCA are shown by a red cross; genes downregulated by TUDCA are shown by a red downward arrow. Proposed mechanisms of action of TUDCA include: inhibition of the intrinsic mitochondrial apoptotic pathway, through reduction of ROS and inactivation of BAX, in turn decreasing cytochrome* c* release; inhibition of the death receptor in the extrinsic apoptotic pathway, with further block of caspase 3; reduction of ER-mediated stress by decreasing caspase 12 activity and Ca^2+^ efflux decrease from the ER. TUDCA also inhibits the apoptotic induced pathways MAPK, JNK, PI3K, NF-кB, ERK and p38 [27–29]. Furthermore, TUDCA is supposed to reduce the expression of genes involved in cell cycle regulation (Cyclin D1), the Apaf-1 apoptotic pathway, and the E2F/p53/BAX, and AP-1 pathways [30, 31]. Abbreviations: AKT, protein kinase B; AP1, activating Protein-1; Apaf-1, apoptotic protease activating factor-1; ATF2, activating transcription factor 2; BAK, Bcl-2 homologous antagonist killer; BAX, Bcl2-associated X protein; Bcl-2, B-cell lymphoma 2 family of regulator proteins; BID, BH3 interacting-domain death agonist C, cytochrome C; CDC42, cell division control protein 42; E2F-1, E2 promoter binding factor 1; ERK, extracellular signal-regulated kinase; IKK-α, nuclear factor kappa-B kinase subunit alpha; IKK-β, nuclear factor kappa-B kinase subunit beta; IKK-γ, nuclear factor kappa-B kinase subunit gamma; JNK, c-Jun N-terminal kinase; KRAS, Kirsten rat sarcoma viral oncogene; MAPKs, mitogen-activated protein kinase; MOM, mitochondrial outer membrane; mTOR, mammalian target of rapamycin; NF-ĸB, nuclear factor kappa B; P, phosphate; p53, cellular tumour antigen p53; PI3K, phosphoinositide 3-kinases; RAC, Rho family of GTPases; RAS, rat Sarcoma Virus; SRF, serum response factor; tBID, truncated BID; TCF, transcription factor; TLR, Toll-like Receptor; TUDCA, tauroursodeoxycholic acid

The use of TUDCA in neurodegenerative diseases is case-dependent. Programmed cell death and apoptosis, a hallmark of all neurodegenerative diseases, share common mechanisms that are targeted by TUDCA. Such mechanisms include endoplasmic reticulum (ER) stress and accumulation of mutated or misfolded protein aggregates. A detailed diagrammatic scheme of TUDCA’s anti-apoptotic effect is outlined in Fig. 2. Most of the pathways on which UDCA and TUDCA exert their anti-apoptotic actions have been extensively studied and characterized in hepatocytes; similar mechanisms are also present in other cell types, including neurons [25, 26].

Furthermore, each disorder is characterized by an underlying context-dependent mechanism, which is also variably targeted by TUDCA. In general, this compound exerts its effects by (i) producing an anti-neuroinflammatory action, (ii) reducing oxidative stress, (iii) regulating and inhibiting the apoptotic cascade, (iv) protecting mitochondria and (v) acting as a chemical chaperone to maintain the stability and correct folding of proteins.

### Gut microbiota

Current research posits the gut microbiota as a possible mediator of the pathological alterations observed in AD, PD and ALS [32]. Gut dysbiosis may augment lipopolysaccharides, pro-inflammatory cytokines, T helper cells and monocytes, causing increased intestinal and BBB permeability via the microbiota-gut-brain axis [33]. Consequently, accumulation of misfolded proteins, axonal damage and neuronal demyelination occur, facilitating the pathogenesis of neurodegenerative disorders.

Biliary acids may influence each of the following three mechanisms through which interactions within the brain-gut-microbiota axis take place: neurological, immunological, and neuroendocrine. These microbial metabolites can act as direct neurotransmitters or neuromodulators, serving as key modulators of the brain-gut interactions. The gut microbial community, through their capacity to produce bile acid metabolites distinct from the liver, can be thought of as an “endocrine organ” with potential to alter host physiology, perhaps to their own favour [34]. Gut dysbiosis may be an important factor in the pathogenesis of neurodegenerative diseases with a concomitant decrease in secondary biliary acid level. Intestinal inflammation due to dysbiosis is related to an increase in proinflammatory cytokines in the circulation, resulting in systemic inflammation. As a result of the concurrent BBB impairment, systemic inflammation may lead to neuroinflammation, a hallmark of many neurodegenerative disorders [35].

While there is evidence of a direct crosstalk between bile acids and gut microbiota, there are also two indirect routes that include intermediary compounds released  upon interactions with bile acid receptors in the gut. When the farnesoid X receptor (FXR) and Takeda G protein-coupled receptor 5 (TGR5) in the gut are activated, fibroblast growth factor 19 and glucagon-like peptide 1 are released, both of which can communicate to the central nervous system. Additionally, bile acids might also be synthesized in the brain [11].

Hydrophilic bile acids, currently regarded as important hormones, exert modulatory effects on the gut microbiota composition to produce secondary bile acids which seem to bind a number of receptors with a higher affinity than the primary biliary acids, expressed on many different cells. Studies suggest that the bile pool size regulates the gut microbiota, ultimately affecting the direct and indirect pathways. Furthermore, alterations of the microbiome-bile acid axis may reduce the risk or progression of certain diseases [35, 36].

### Evidence from animal models

There are many studies focusing on multiple pathophysiological and cellular aspects of TUDCA in AD, HD, PD, ALS, and brain ischemia.

### Alzheimer’s disease models

Animal models of AD are based on genetic and proteomic alterations that are known to underlie the clinical presentation of the disease. The neuropathological hallmarks of AD are: amyloid plaque deposits, fibrillary tangles and chronic neuroinflammation. The spectrum of evidence for TUDCA ranges from in vitro studies on primary neuronal cultures and neuroblastoma cell lines (either incubated with amyloid-β or expressing genetic mutations of AD-associated genes) to in vivo studies on transgenic AD murine models carrying mutations in the amyloid precursor protein (APP), presenilin 1 (PS1) or presenilin 2 (PS2) genes.

In vitro studies incubating primary rat neurons or PC12 neuronal cells with amyloid-β showed increased levels of apoptosis, which were prevented by TUDCA [37, 38]. In particular, TUDCA was shown to modulate the amyloid-β-induced apoptosis by interfering with upstream targets of the apoptotic mitochondrial pathway, including the E2F-1/p53/Bax pathway [39]. A study in cortical neurons confirmed that the amyloid-β-induced apoptosis proceeds through the Bax mitochondrial pathway and that the PI3K signalling cascade plays a central role in regulating the anti-apoptotic effects of TUDCA [40]. TUDCA was also found to interact with GSK3β, an imperative key player in tau hyper-phosphorylation and glial activation, and to play a role in the modulation of the apoptotic Akt signalling pathway [41].

As an in vitro model of familial AD, mouse neuroblastoma cells expressing either wild-type APP, APP with the Swedish mutation or the double-mutated human APP and PS1, showed that familial AD mutations are associated with the activation of classical apoptotic pathways [42]. By contrast, TUDCA reduced nuclear fragmentation and the activity of caspases 2 and 6. The expression of Bcl-2 and Bax and the activity of p53 were also modulated by TUDCA in this model [42]. Other evidence in favour of TUDCA modulation of amyloid-β-induced toxicity was obtained in primary human cerebral endothelial cells incubated with the vasculotropic E22Q Amyloid-β (AβE22Q) mutant, which is associated with hereditary cerebral haemorrhage in Dutch type amyloidosis [43]. This study revealed that AβE22Q triggered the Bax mitochondrial pathway of apoptosis, an effect that was modulated by TUDCA. Interestingly, within the same study, TUDCA was unable to decrease the secondary structure and the fibrillogenic propensities of amyloid-β, suggesting a dissociation between the pro-apoptotic properties of amyloid-β peptides and their distinct mechanisms of aggregation and fibrillization in vitro*.*

Due to their potential role in amyloid-β-induced toxicity, neuronal mitochondria were isolated and co-incubated with TUDCA, to further assess its protective effect against apoptosis. Amyloid-β was found to induce oxidative injury and profound structural changes on mitochondrial membranes, including modified membrane lipid polarity and disrupted protein mobility; as a result of the increased membrane permeability, cytochrome *c* was released from the inter-membrane space of mitochondria [44]. Co-incubation with TUDCA almost completely abolished the amyloid-β-induced perturbation of mitochondrial membrane structure and consequent cytochrome *c* release in isolated mitochondria [37]. Moreover, using electron paramagnetic resonance spectroscopy analysis, it was demonstrated that TUDCA prevented the amyloid-β-driven modifications of the mitochondrial membrane redox status, the lipid polarity, and the structure of superficial mitochondrial membrane proteins [44].

TUDCA has also been shown to mitigate the toxic downstream effects of amyloid-β. TUDCA inhibits the levels of apoptosis and caspase-3 activation, and abolishes the caspase-3 cleavage of tau into a toxic species in primary rat cortical neurons incubated with fibrillary amyloid-β 1–42 [45]. Cleavage of tau by caspase-3 at Asp421 in the C-terminal region is linked to increased aggregation of tau filaments, and can be detected both in transgenic AD mouse models and in the brains of patients affected by AD [46]. Thus, by interfering with apoptotic pathways, at both the mitochondrial and transcriptional levels, TUDCA seems not only to increase the survival of neurons, but also to prevent the downstream abnormal conformations of tau.

Growing evidence supports inhibition of the unfolded-protein response (UPR) as another possible mechanism underlying the neuroprotective actions of TUDCA. TUDCA acts as a molecular chaperone, ameliorating ER stress and preventing UPR dysfunction by improving protein folding capacity [47]. Although the exact mechanism of its chaperoning activity is still unclear, it has been shown that TUDCA exerts these effects by assisting in the transfer of mutant proteins via the activation of transcription factor 6 in various cell types [48]. In keeping with it, TUDCA has been shown to prevent tau hyperphosphorylation via inhibition of the UPR in human neuroblastoma cell lines [49]. Moreover, TUDCA administration to a transgenic mouse model of familial amyloidotic polyneuropathy significantly reduces transthyretin toxic aggregates, in turn decreasing apoptotic and oxidative biomarkers that are usually associated with transthyretin deposition [50].

TUDCA is able to exert a protective effect also at the synaptic level of deranged neurocircuitry. One of the earliest hallmarks of neurodegeneration is synaptic loss. TUDCA has been shown to reduce the downregulation of the postsynaptic density-95 protein, to decrease spontaneous miniature excitatory synaptic activity and to increase the number of dendritic spines in a mouse model of AD [51]. This remarkable effect of TUDCA at the synaptic level suggests that the neuroprotective role of this bile acid is not limited to neuronal survival, but can possibly be extended to a restoration of the synaptic function.

Concerning in vivo studies, TUDCA significantly attenuates amyloid-β deposition in the brain and decreases amyloid-β 1–40 and 1–42 levels in transgenic APP/PS1 AD mice, suggesting reduced amyloidogenic production [41]. Importantly, in the same study, TUDCA portrayed anti-inflammatory properties, by modulating glial activation and mRNA expression of cytokines [41]. Finally, TUDCA supplementation prevents cognitive impairment in APP/PS1 transgenic AD mice, which display intact spatial recognition and contextual memory, together with a general reduction in amyloid deposition in the hippocampus and prefrontal cortex [52].

In summary, data from AD animal models suggest that TUDCA may have a disease-modification role in AD progression. Although further studies are required to show a disease-modifying effect in humans, the potential interactions of TUDCA with the apoptotic cascade leading to disease progression at neuronal level are also highlighted in AD animal models. Further characterization of these signalling pathways and of the exact targets is likely to provide new perspectives for modulation of amyloid-β-induced apoptosis by TUDCA.

### Huntington’s disease models

The neuroprotective role of bile acids has been explored also in the 3-nitropropionic acid (3-NP) model of HD. Systemic administration of 3-NP, an irreversible inhibitor of succinate dehydrogenase and of the mitochondrial citric acid cycle, produces selective striatal degeneration. This model is reminiscent of the neurochemical and anatomical changes associated with HD, and also shows that higher doses of 3-NP compare to more advanced HD clinical stages [53].

In cultured neuronal cells incubated with 3-NP, TUDCA markedly reduces the mitochondrial perturbations that are associated with apoptosis induction [54]. Consistently, the systemic administration of TUDCA (50 mg/kg) in a 3-NP rat HD model reduces the associated morphologic striatal lesions [8]. Moreover, behavioural studies correlated with the histopathological findings, since neuroprotection resulted in the near prevention of hyperactive behaviour in the Rota Rod performance test. In addition, rats receiving a combination of TUDCA and 3-NP had comparable performance in sensorimotor and cognitive tasks to that of non-treated controls, and this effect persisted for at least 6 months [8].

These initial studies were extended to the R6/2 transgenic mouse model of HD, which began receiving TUDCA at 6 weeks of age. Results showed reduced striatal atrophy, decreased striatal apoptosis, as well as fewer and smaller ubiquitinated neuronal intranuclear “huntingtin” inclusions in these mice with TUDCA treatment. Moreover, locomotor and sensorimotor deficits were significantly improved in the TUDCA-treated mice [25].

### Parkinson’s disease models

PD can be modelled in animals by administering neurotoxic drugs, by disrupting neurotransmitter release, or by manipulating genes associated with the familial forms of the disease [55]. Using these models*,* it has been possible to reproduce and further characterize the activity of TUDCA on the cellular pathways possibly involved in the onset of PD.

TUDCA was shown to improve the survival and function of nigral transplants in rats subjected to 6-hydroxydopamine lesioning of the mesostriatal dopamine system [56]. Indeed, TUDCA, at an undocumented dosage, significantly reduced apoptosis in ventral mesencephalic tissue cultures and within the transplants. This suggested that the bile acid may exert beneficial effects on dopamine neuronal survival, mainly through neuronal death inhibition. The number of apoptotic cells was in fact much lower in the graft areas of the TUDCA-treated groups, when compared to the control group 4 days after transplantation. These data demonstrate that pre-treatment of the cell suspension with TUDCA can reduce apoptosis and increase the survival of nigral grafted cells, resulting in an improvement of behavioural recovery.

In a *Caenorhabditis elegans* model of PD obtained by genetically manipulating three mitochondrial proteins that are mutated in monogenic PD (α-synuclein, DJ-1 and parkin), TUDCA was shown to ameliorate the pathological phenotype by exerting a protective action on mitochondria [57]. Moreover, TUDCA has been shown to be effective in preventing the 1-methyl-4-phenyl-1,2,3,6-metrahydropyridine (MPTP)-induced neurodegeneration in mice through reduction of JNK phosphorylation and ROS production, and upregulation of glutathione S-transferase catalytic activity and Akt signalling pathway [58]. Additionally, TUDCA increases the expression of Nrf2, Nrf2-stabilizer DJ-1, and Nrf2 downstream target antioxidant enzymes (HO-1 and GPx) in mice. This suggests that TUDCA, at a dose of 50 mg/kg, can possibly alleviate ROS-mediated apoptosis in animal models of PD [59].

A more recent study has confirmed that pre-treatment with TUDCA can prevent mitochondrial dysfunction and neuronal death in the MPTP mouse model [60]. Moreover, modulation of parkin phosphorylation at Ser65 causes activation of mitophagy, which can be hindered by TUDCA pre-treatment (50 mg/kg). The study portrayed a possible role of TUDCA on PINK1/parkin-mediated pathway, which underlies its neuronal protective effect [60]. In another study on the same PD model, the authors reported that TUDCA prevents the MPTP-dependent decrease of dopaminergic fibers and ATP levels, mitochondrial dysfunction and neuroinflammation. Following TUDCA administration (50 mg/kg), the mice also displayed reduction in foot dragging and an overall improvement in gait [61]. These observations suggest TUDCA as a prophylactic treatment in animal models, shedding light on therapies in translational preventative medicine for risk groups.

Another study in the MPTP mouse model of PD also highlighted an anti-neuroinflammatory potential of TUDCA. This bile acid was shown to reduce the pro-inflammatory cytokine interleukin 1 beta and markers of astro- and microgliosis, while increasing the level of the anti-inflammatory protein Annexin-A1 [62]. These data suggest a possible link between suppression of neuroinflammation by TUDCA and neuroprotection, which deserves further characterization in future studies.

### Amyotrophic lateral sclerosis models

Motor neuron death in ALS has been attributed to oxidative damage, axonal strangulation from intracellular aggregates and glutamate excitotoxicity [63, 64]. Genetic factors, changes in intracellular calcium levels in motor neurons, and programmed cell death have also been linked to the development of ALS [65, 66]. In addition, evidence suggests a role for mitochondrial and energy dysfunction in the pathogenesis of ALS [67]. As in other neurodegenerative diseases, abnormal protein deposits are also observed [65]. Animal models of ALS are based on genetic alterations that are known to cause the human disease. In particular, mice with mutations in the superoxide dismutase 1 (SOD-1) gene hold importance in constructing insights into the pathogenesis of ALS in humans [68]. Genetically determined ALS accounts only for 5%–10% of all ALS cases, which mostly include sporadic presentations; mutations in the SOD-1 gene are found in up to 30% of familial cases (2%–3% of total patients) [66]. Despite the small percent in the entire spectrum of human ALS, the SOD-1 ALS mouse model has provided a first breakthrough in uncovering the pathogenic features of motor neuron degeneration in ALS [68]. Features such as changed cell morphology, caspase activation, altered balance between pro- and anti-apoptotic molecules and redistribution of these molecules from the cytosol to the mitochondria have been observed also in this and other ALS murine models [69]. The identification of a final common pathway may lead to the development of novel treatment modalities.

Accordingly, the mouse ALS model has been used to test the potential neuroprotective effect of bile acids. Mice carrying the ALS-causing G93A mutation in the human SOD1 gene were compared to those expressing the wild-type human SOD1 gene. Both groups were treated with TUDCA 0.5 mg/g every 3 days, for a total of 7 injections [70]. The results showed an increase in neuromuscular junction innervation in the mutated mice following treatment with TUDCA. This effect was confirmed also on cultured human motor neurons carrying the G93A SOD1 mutation, which displayed strong neurite outgrowth after treatment with TUDCA [70].

These models have unravelled new possible pathogenic mechanisms of ALS at the cellular level. Further exploitation of these experimental models is needed to uncover the molecular and cellular factors underlying the potential therapeutic actions of TUDCA in ALS.

### Cerebral Ischemia models

The anti-degenerative properties of TUDCA have also been investigated in acute conditions, particularly in rat models of transient focal cerebral ischemia. In a model of middle cerebral artery occlusion, experimental ischemia was found to induce mitochondrial swelling and caspase activation [26]. TUDCA administration 1 h after ischemia resulted in significantly increased bile acid levels in the brain, improved neurologic function, and ~ 50% reduction in infarct size, as assessed 2 and 7 days after reperfusion. In addition, TUDCA significantly reduced mitochondrial swelling, and partially inhibited caspase-3 processing and substrate cleavage [26]. These findings suggest that the mechanisms of in vivo neuroprotection by TUDCA are, at least in part, mediated by inhibition of mitochondrial dysfunction and consequent energetic deficit that triggers caspase activation, subsequently leading to cell death.

Importantly, it has been demonstrated that intravenous administration of TUDCA reduces the infarct volume, modulates the levels of apoptosis, and inhibits the neurobehavioral impairment in a collagenase-induced haemorrhagic model of stroke [9]. The administration of TUDCA before or up to 6 h after stereotaxic collagenase injection into the striatum reduced lesion volumes at 2 days by as much as 50%. The apoptosis was decreased by ~ 50% in the area immediately surrounding the hematoma and was associated with a similar inhibition of caspase activity. These changes were also associated with improved neurobehavioral deficits, as assessed by rotational asymmetry, limb placement, and stepping ability tests. Furthermore, TUDCA treatment modulated the expression of certain Bcl-2 family members, as well as nuclear factor kappa B (NF-ĸB) activity. In addition to its protective action at the mitochondrial membrane, TUDCA also activated the Akt survival pathway and induced Bad phosphorylation at Ser136 [9].

TUDCA treatment (100 mg, 3 times/day) also reduces neurological impairment in rats with acute cerebral infarction. The authors speculated that TUDCA can alter lipid peroxidation in the inflammatory response, therefore decreasing apoptosis through the Nrf2 signalling pathway and the inhibition of caspase and mitochondrial apoptotic pathways [71].

### Evidence on human diseases

Overall, animal disease models have provided substantial evidence for the therapeutic properties of TUDCA in halting apoptotic pathways. Although AD, HD, PD, ALS, and cerebral ischemia have different disease progressions, they share similar pathways which can be targeted by TUDCA. This makes this bile acid a potentially strong therapeutic option to be tested in human diseases. Clinical evidence collected so far has reported comprehensive data on ALS only. Regarding other neurodegenerative diseases, there are only scattered data and clinical trials are currently underway (Table 1). It is not yet timely to review data on AD, PD, stroke and HD. We focus this review on published clinical data that regard ALS and allow for detailed analysis and innovative conclusions.

**Table 1:** Registered clinical trials on hydrophilic bile acids in different neurodegenerative diseases

Intervention(s)	Condition	Dose	Duration	Phase	Study design	Status	References
UDCA	ALS	15, 30, or 50 mg/kg per day	1 month	I	Open label	Published	[10]
UDCA versus Placebo	ALS	3.5 g/140 ml per day	3 months	III	Randomized, Double blind, Crossover	Published	[88]
TUDCA versus Placebo	ALS	2 g/day	13.5 months	II	Randomized, Double blind, Parallel arm	Published	[89]
TUDCA + NaPB versus Placebo	ALS	2 g/day TUDCA + 6 g/day NaPB	4 months	II	Randomized, Double blind, Parallel arm	Published	[90]
UDCA versus Placebo	HD	600 or 1200 mg/day	1 month	I	Randomized, Double blind, Parallel arm	Unknown	NCT00514774
TUDCA versus Placebo	ALS	2 g/day	18 months	III	Randomized, Double blind, Parallel arm	Recruiting	NCT03800524
TUDCA + NaPB	ALS	2 g/day TUDCA + 6 g/day NaPB	up to 30 months	N/A	Open label	Enrolling by invitation	NCT03488524
TUDCA versus Placebo	MS	2 g/day	4 months	I/II	Randomized, Double blind, Parallel arm	Recruiting	NCT03423121
TUDCA + NaPB versus Placebo	AD	2 g/day TUDCA + 6 g/day NaPB	6 months	II	Randomized, Double blind, Parallel arm	Active, not recruiting	NCT03533257
UDCA	PD	50 mg/kg per day	1.5 months	I	Open label	Not yet recruiting	NCT02967250
UDCA versus Placebo	PD	30 mg/kg per day	12 months	II	Randomized, Double blind, Parallel arm	Active, not recruiting	NCT03840005
TUDCA + NaPB	ALS	2 g/day TUDCA + 6 g/day NaPB	6 months	II	Open label	Enrolling by invitation	NCT04516096
TUDCA + NaPB versus Placebo	ALS	2 g/day TUDCA + 6 g/day NaPB	12 months	III	Randomized, Double blind, Parallel arm	Not yet recruiting	NCT05021536

*AD* Alzheimer's disease, *ALS* amyotrophic lateral sclerosis, *HD* Huntington's disease, *N/A* not applicable, *MS* multiple sclerosis, *NaPB* sodium phenylbutyrate, *PD* Parkinson's disease, *TUDCA* tauroursodeoxycholic acid, *UDCA* ursodeoxycholic acid

### Safety of hydrophilic bile acids

Safety of experimental compounds is of paramount importance for clinical testing in humans. A wide experience on the safety of hydrophilic bile acids has been obtained on hepatobiliary indications. UDCA and TUDCA have been used in the treatment and prevention of cholesterol gallstones and in a variety of conditions, such as primary biliary cirrhosis, liver cirrhosis, primary sclerosing cholangitis, chronic hepatitis C, polycystic liver disease, intrahepatic cholestasis of pregnancy and fatigue in chronic liver disease (Table 2). All these studies have generally confirmed a good safety profile, reporting mostly mild gastrointestinal adverse events, particularly diarrhoea, abdominal pain, nausea and vomiting, and less frequently rashes and pruritus.

**Table 2:** Safety data on hydrophilic bile acids arising from the available human studies

Intervention(s)	Condition	Dose	Duration	Number of participants	Age	Safety findings	References
UDCA	Gallstones	12 mg/kg per day	3 months	34	18–80 years	Well tolerated. No side effects were reported	[72]
UDCA	Gallstones	300 or 600 mg/day	13 months	20	> 18 years	Well tolerated. The most common adverse events were nausea and skin rash	[73]
UDCA + CDCA, UDCA	Gallstones	10 mg/kg per day	24 months	596	> 20 years	Both interventions were well tolerated. The most common adverse events were diarrhoea, abdominal pain, nausea, vomiting, dizziness and asthenia	[74]
UDCA	Primary biliary cirrhosis	300 mg/day	3 months	24	18–75 years	Well tolerated. Two patients had severe diarrhoea, and terminated the trial prematurely	[75]
UDCA	Primary biliary cirrhosis	250 or 500 mg/day	3 months	199	18–70 years	Well tolerated. The most common adverse events were abdominal pain, eructation, abdominal distension, nausea, and vomiting	[76]
UDCA	Primary biliary cirrhosis	15 mg/kg per day	24 months	184	> 19 years	Well tolerated. The most common adverse events were abdominal pain, flatulence and diarrhoea	[77]
TUDCA	Primary biliary cirrhosis	500, 1000 or 1500 mg/day	6 months	159	18–75 years	Well tolerated. Three patients had severe diarrhoea, and terminated the trial prematurely	[78]
UDCA, TUDCA	Primary biliary cirrhosis	750 mg/day	6 months	154	> 18 years	Both interventions were well tolerated. The most common adverse events were diarrhoea, pruritus, rash and dysmenorrhoeal in the TUDCA group, rash and nausea in the UDCA group	[79]
UDCA, TUDCA	Primary biliary cirrhosis	500 mg/day	6 months	192	18–72 years	Both interventions were well tolerated. No side effects were reported	[80]
UDCA	Primary sclerosing cholangitis	20 mg/kg per day	5 years	30	> 18 years	The most common adverse events were diarrhoea, loose stools, pruritus, anorexia and flatulence	[81]
norUDCA	Primary sclerosing cholangitis	500, 1000 or 1500 mg/day	3 months	219	18–70 years	Well tolerated at all doses, except by two patients in the 1000 mg group	[82]
UDCA	Chronic hepatitis C	150, 600 or 900 mg/day	6 months	65	> 18 years	Well tolerated. The most common adverse events were diarrhoea	[83]
UDCA, TUDCA	Liver cirrhosis	750 mg/day	6 months	625	> 18 years	Well tolerated. No side effects were reported	[84]
UDCA	Polycystic liver disease	20 mg/kg per day	6 months	23	18–75 years	The most common adverse events were frequent stools or diarrhoea	[85]
UDCA	Intrahepatic cholestasis of pregnancy	450 mg/day	14 days	23	18–70 years	No adverse events during or after the treatment	[86]
UDCA	Fatigue in chronic liver disease	Not reported	Not reported	20	Not reported	Well tolerated. No side effects were reported	[87]
UDCA	ALS	15, 30, or 50 mg/kg per day	1 month	30	> 18 years	Well tolerated. The most common adverse events were gastrointestinal	[10]
UDCA	ALS	3.5 g/140 mL per day	3 months	65	> 18 years	Well tolerated. The most common adverse events were gastrointestinal	[88]
TUDCA	ALS	2 g/day	13.5 months	20	Not reported	Well tolerated. The most common adverse events were mild diarrhoea and anorexia	[89]
TUDCA + NaPB	ALS	2 g/day TUDCA + 6 g/day NaPB	4 months	23	18–75 years	Well tolerated. The most common adverse events were nausea, diarrhoea, and abdominal pain	[90]

*ALS* Amyotrophic Lateral Sclerosis, *CDCA* chenodeoxycholic acid, *NaPB* Sodium phenylbutyrate, *norUDCA* Norursodeoxycholic acid, *TUDCA* tauroursodeoxycholic acid, *UDCA* ursodeoxycholic acid

More recently, bile acids have been administered to patients with ALS. In a pilot study, UDCA was administered to 18 ALS patients randomly assigned to receive 15, 30 or 50 mg/kg UDCA daily for 4 weeks [10]. UDCA was well tolerated by all subjects at all doses. The most common adverse events were gastrointestinal, including constipation, loose bowel movements, nausea, and abdominal bloating. An oral soluble UDCA formulation (3.5 g/140 ml per day) was instead tested for three months in 64 ALS patients in a crossover trial [88]. Apart from the expected complications of ALS, such as dyspnoea and dysphagia, adverse events that could be possibly attributed to the study drug were reported in 12 patients (16.2%) treated with UDCA and in 6 patients (8.6%) treated with placebo. These events were mostly gastrointestinal and developed more frequently when patients were treated with UDCA.

Similar findings have been reported for TUDCA. A phase II double-blind placebo-controlled study evaluated the safety and efficacy of 2 g daily TUDCA in ALS patients for 54 weeks [89]. The population for safety analysis consisted of 15 patients who took TUDCA and 14 patients who took placebo. The treatment was well tolerated in all patients. Apart from the ALS-related adverse events, no changes in vital signs and laboratory values that could possibly be attributed to the study drug or placebo were recorded. Overall, five adverse reactions were blindly considered by the investigators to be related to the study medication: mild diarrhoea that occurred in two patients treated with TUDCA and in two treated with placebo, and anorexia that was reported in a placebo-treated patient.

A recent phase II trial randomized 137 ALS patients to an oral co-formulation of 1 g TUDCA and 3 g sodium phenylbutyrate (NaPB) or matching placebo twice daily for 24 weeks [90]. Gastrointestinal adverse events were reported more frequently in the active-drug group than in the placebo group during the first 3 weeks, with nausea, diarrhoea, and abdominal pain being the most frequent events. Their frequency decreased in both groups for the remaining 21 weeks. The most common adverse events leading to discontinuation of the treatment were diarrhoea (6% in the active group and none in the placebo group) and respiratory failure (6% in the placebo group and none in the active group).

### Efficacy on neurodegenerative diseases

Although preclinical evidence on the neuroprotective action arises from animal models of different neurodegenerative conditions, hydrophilic bile acids have so far been tested almost exclusively on patients affected by ALS. However, recent trials have started investigating the bile acids on other conditions, such as AD, PD and multiple sclerosis. Table 1 lists the clinical studies assessing hydrophilic bile acids in neurodegenerative conditions.

ALS represents a unique environment to investigate the potential usefulness of hydrophilic bile acids as a mainstay therapy. ALS is a fast-progressing neurodegenerative disease, with minimal treatment options that have shown a significant degree of efficacy towards modifying the disease course [63, 64]. Since there is evidence for a derangement of the apoptotic pathways in ALS [66, 69], it can be hypothesized that the administration of TUDCA to ALS patients could improve symptoms of the disease and possibly halt or slow down its currently invariable progression.

The pilot study administering three different doses of UDCA (15, 30 or 50 mg/kg) to 18 ALS patients for 4 weeks provided relevant findings on the pharmacokinetic profile and central nervous system penetration of the drug, showing for the first time in humans that UDCA crosses the BBB in a dose-dependent manner [10].

The efficacy of oral solubilized UDCA (3.5 g/140 ml per day) on ALS patients was also assessed in a longitudinal crossover study, comparing UDCA and placebo treatment for 3 months, with a 1-month washout between the crossover phases [88]. The primary outcome was the Appel ALS rating scale: in the UDCA-treated group the score was 1.17 points/month lower in comparison to controls (*P* = 0.037). No significant between-group difference was found for the secondary outcome measure (revised ALS functional rating scale, ALSFRS-R). This preliminary observation was focused on a possible beneficial effect of UDCA on functional decline in ALS. Although disease progression could benefit from this treatment, the study design had relevant limitations, such as the high drop-out rate, a short treatment duration, and unequal randomization (40 patients taking the treatment in comparison to 23 as controls on placebo). Therefore, a conclusive decision could not be made.

The efficacy of TUDCA as an add-on to riluzole in patients with ALS was reported for the first time in a pilot phase II study [89]. This trial had a double-blind design, included patients with spinal-onset ALS and was less than 18 months of disease course. At the time of enrollment, patients had a forced vital capacity > 75% and were on a steady riluzole regimen. This study included a no-treatment run-in period of 12 weeks, which allowed measuring the natural disease progression rate before starting the experimental treatment. The patients then received 1 g of TUDCA twice daily or placebo for 54 weeks. Responder patients were defined as those showing a ≥ 15% difference in the decline of the ALSFRS-R slope between run-in and treatment periods. The study showed convincing evidence to conclude that there was a halting effect on the disease progression from the administration of TUDCA, which was additive to that of riluzole. The percentage of responding patients was significantly higher in the TUDCA arm than in the placebo arm (*P* = 0.021). The baseline-adjusted ALSFRS-R score was significantly higher in TUDCA-treated than in placebo-treated patients (*P* = 0.007). The per year decline rate on the ALSFRS-R was 7-point smaller (on a 0–48 range) for patients treated with TUDCA compared to those treated with placebo. The study reported the average survival time of 65.7 weeks with TUDCA, and 61.1 weeks with placebo. Unfortunately, this study observed no difference in forced vital capacity, whose decline is associated with poor prognosis in ALS.

A second phase II study tested the efficacy of a combination of TUDCA and NaPB. This randomized double-blind study assigned 89 patients to TUDCA + NaPB and 48 to placebo. The study’s functional assessment similarly followed the ALSFRS-R score and showed a difference (*P* = 0.03) in the mean rate of change between the active drug combination group, as opposed to placebo. This indicated a slower functional decline in disease progression over the study period of 24 weeks. This study also shed light on secondary outcomes in ALS patients that progressed to a morbid demise, such as the rate of decline in isometric muscle strength, breathing function, or the time to tracheostomy, permanent ventilation, and hospitalization, which showed no variable difference between the two study groups [90]. This study also reported no difference between groups in plasma phosphorylated axonal neurofilament H subunit, an important biomarker for axonal degeneration.

### Monotherapy *versus* combination therapy

**Fig. 3:**
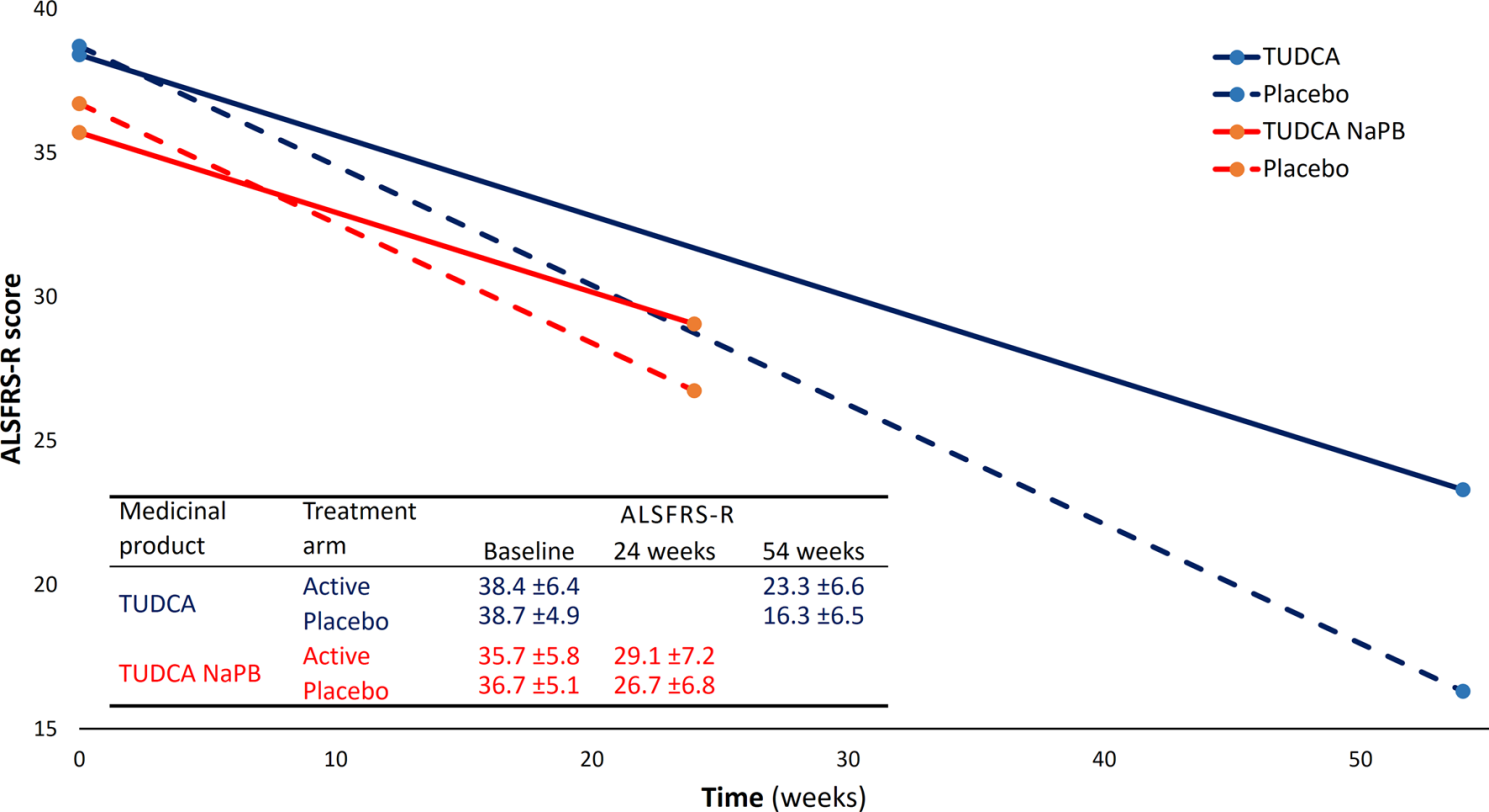
ALSFRS-R functional decline reported by phase II studies on TUDCA in ALS. The mean reported decline is plotted for the study on TUDCA alone (blue) [89] and for TUDCA and NaPB study (red) [90]. Solid lines indicate the active groups; dashed lines represent the control groups. The table reports means (± SD) for baseline and end-of-study measures [89, 90]. Abbreviations: ALSFRS-R, Amyotrophic Lateral Sclerosis Functional Rating Scale-Revised; NaPB, sodium phenylbutyrate; TUDCA, tauroursodeoxycholic acid

The two recent phase II studies on bile acids in ALS [89, 90] highlight TUDCA and NaPB as potential disease-modifiers in ALS and raise the question of whether there is added value in administering a combination of these compounds versus their use in monotherapy. Both studies used ALSFRS-R as the primary outcome measure and the baseline ALSFRS-R scores were similar (Fig. 3). The two studies differed instead mainly in the duration of treatment (54 weeks for TUDCA versus 24 weeks for TUDCA and NaPB) and sample sizes (34 patients with 1:1 randomization for TUDCA versus 137 patients with 2:1 randomization for TUDCA and NaPB) [91].

NaPB is a histone deacetylase inhibitor that is believed to attenuate ER stress by acting as a chaperone [92]. NaPB has been shown to reduce neuronal death in experimental models of neurodegenerative diseases, including ALS [93, 94]. A pilot open-label trial reported the safety and tolerability of NaPB in 40 patients with ALS but did not observe efficacy of this compound [95]. However, the study was not specifically designed to detect a disease-modifying activity. TUDCA and NaPB were directly compared in a mouse model of type 2 diabetes, where they showed similar capability to reduce ER stress [96]. There are currently no planned clinical trials on NaPB monotherapy for ALS or other neurodegenerative diseases.

While both TUDCA and NaPB individually have neuroprotective action in experimental models, preliminary evidence of disease-modifying potential has been shown only for TUDCA. Furthermore, it remains to be understood whether a combination of the two compounds may yield additional potential on ALS. Stronger premises would be warranted for usage of the TUDCA + NaPB co-formulation in humans.

When comparing the two available phase II clinical trials on TUDCA in ALS, there is remarkable resemblance in the outcome reported by TUDCA alone [89] and by the TUDCA + NaPB combination [90]. In both studies, the ALSFRS-R progression slopes were milder in the treated groups. Remarkably, the control groups and the treated groups of these studies had parallel progression trajectories, suggesting that TUDCA is the main or only disease-modifier in ALS (Fig. 3).

Whether TUDCA, NaPB, and a combination of the two are efficacious in ALS remains at present a very relevant open question. Given the fact that there is no planned study on NaPB alone in ALS, the answer fundamentally lies within the ongoing phase III TUDCA trial. This is a European Union-funded study to test the safety and efficacy of TUDCA in patients affected by ALS (NCT03800524). The primary aim of the trial is to measure the proportion of responding patients in TUDCA and placebo arms by the ALSFRS-R. Furthermore, survival time, quality of life, treatment safety and tolerability, and other functional assessments, such as forced vital capacity or muscle force, are also planned. The efficacy of TUDCA will also be tested by measuring biomarkers of disease progression, such as neurofilaments and matrix metalloproteinase-9, hopefully providing additional information on the biological aspects of the neuroprotective action of TUDCA. Although no studies on NaPB alone are planned, a large multicentre trial of TUDCA and NaPB is designed to start soon (NCT05021536). Further studies that delineate NaPB as a monotherapy for neurodegenerative diseases need to be carried out to unravel whether it has a disease-modifying clinical outcome and whether this is additional to that of TUDCA.

## Conclusion and outlooks

The evidence reporting the beneficial effects of hydrophilic bile acids in animal models of different neurodegenerative diseases, as well as the preliminary results from clinical trials in ALS, indicates TUDCA as a candidate with a great disease-modification potential. Although the mechanisms by which TUDCA exerts its neuroprotective effect are not fully understood, there are current hypotheses on action at different cellular levels.

A first consideration is the strict interrelationship between bile acids and the microbiota. The size of the bile acid pool appears to be controlled by the host and microbiota. The size of the bile pool additionally modulates the gut microbiota, altering both direct and indirect pathways. Changes in the microbiome-bile acid axis may decrease the risk of some diseases or slow their course [36, 97].

Studies examining the neuroprotective effect of TUDCA were focused mostly on apoptosis and mitochondrial dysfunction. This is in keeping with data showing that, while hydrophilic bile acids are cytoprotective, hydrophobic bile acids instead promote the apoptotic process. As reported above, it is now believed that the anti-apoptotic effect of TUDCA is achieved through five main mechanisms:

1. inhibition of the intrinsic mitochondrial apoptotic pathway, reducing ROS production, and inhibiting Bax translocation, and consequently cytochrome *c* release [98];
2. inhibition of the extrinsic apoptotic pathway, inhibiting death-receptors and blocking capsase-3 [99];
3. reduction of ER-mediated stress [100], reducing calcium efflux from ER and caspase-12 activity;
4. inhibition and direct modulation of the survival signalling pathways [101–103]; and
5. regulation of the expression of genes involved in cell cycle and apoptotic pathways [104, 105].

**Fig. 4:**
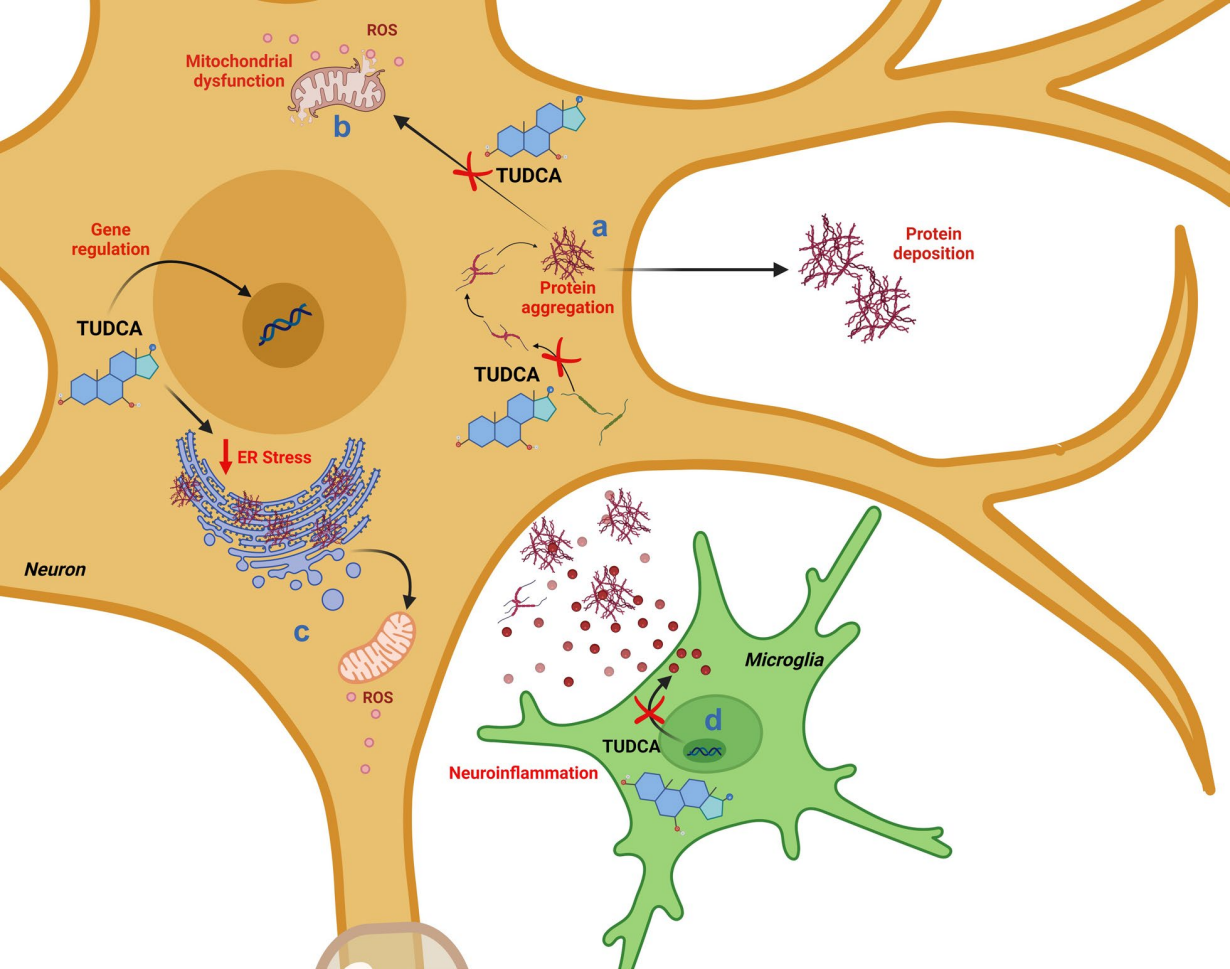
The potential neuroprotective effects of TUDCA are shown in context with deposition of protein aggregates and neuroinflammation. TUDCA regulates the expression of genes involved in cell cycle regulation and apoptotic pathways, thus promoting neuronal survival, as depicted in Fig. 2. TUDCA improves protein folding capacity through its chaperoning activity, in turn reducing protein aggregation and deposition (**a**). By preventing protein aggregation, TUDCA also reduces ROS production, ultimately leading to protection against mitochondrial dysfunction (**b**), and ameliorates ER stress (**c**). Finally, TUDCA inhibits the expression of pro-inflammatory cytokines, in turn exerting an anti-neuroinflammatory effect (**d**)

Apart from its anti-apoptotic action, consistent evidence has shown that the mechanisms by which these bile acids exert their neuroprotective effect may encompass also other pathways involved in neuronal degeneration, such as those involved in protein homeostasis or neuroinflammation, as well as in synaptic function [49, 51, 62] (Fig. 4). Overall, the administration of TUDCA in animals has proven to specifically target the deranged/pathological biochemical pathways underlying cell death and neurodegeneration. Although anti-apoptotic, anti-inflammatory, and several positive effects have been reported for this compound in multiple neurodegenerative conditions, little is known about the prevalent mechanisms underlying neuroprotection induced by TUDCA in each of these conditions. Novel clinical trials are needed to confirm whether TUDCA’s neuroprotective action corresponds to a disease-modifying effect. We foresee that TUDCA may find specific indication in some neurodegenerative conditions where its mechanisms of action are key to produce clinically appreciable disease modification. In efforts to unravel these mechanisms, more research on TUDCA’s neuroprotective and disease-modifying activity is warranted.

## Data Availability

Research data are available in analytical form on the institutional server and can be provided upon request.

## Abbreviations

3-NP: 3-Nitropropionic acid
AD: Alzheimer’s disease
ALS: Amyotrophic lateral sclerosis
ALSFRS-R: Revised ALS functional rating scale
APP: Amyloid precursor protein
BBB: Blood brain barrier
ER: Endoplasmic reticulum
HD: Huntington’s disease
NaPB: Sodium phenylbutyrate
PD: Parkinson’s disease
PS1: Presenilin 1
PS2: Presenilin 2
ROS: Reactive oxygen species
SOD-1: Superoxide dismutase 1
TUDCA: Tauroursodeoxycholic acid
UDCA: Ursodeoxycholic acid
UPR: Unfolded-protein response
